# A Comparison of the Reliability of Five Sleep Questionnaires for the Detection of Obstructive Sleep Apnea

**DOI:** 10.3390/life12091416

**Published:** 2022-09-10

**Authors:** Šárka Solecka, Karel Matler, Tomáš Kostlivý, Vojtěch Kubec, Hana Tomášková, Jaroslav Betka

**Affiliations:** 1 Department of Otorhinolaryngology, Hospital in Frýdek-Místek, 73801 Frýdek-Místek, Czech Republic; 2 Department of Epidemiology and Public Health, Faculty of Medicine, University of Ostrava, 70103 Ostrava, Czech Republic; 3 Department of Otorhinolaryngology, Faculty of Medicine in Pilsen, University Hospital in Pilsen, Charles University, 30000 Pilsen, Czech Republic; 4 Department of Otorhinolaryngology and Head and Neck Surgery, First Faculty of Medicine, University Hospital Motol, Charles University, V Uvalu 84, 15006 Prague, Czech Republic

**Keywords:** obstructive sleep apnea, Berlin questionnaire, STOP-Bang questionnaire, STOP questionnaire, Epworth Sleepiness Scale, Pittsburgh Sleep Quality Index

## Abstract

The aim of this study was to compare the reliability of five sleep questionnaires in detecting the occurrence of obstructive sleep apnea (OSA). The study was conducted on a group of 201 patients. The patients completed five sleep questionnaires: the Epworth Sleepiness Scale (ESS), the STOP-Bang questionnaire, the STOP questionnaire, the Berlin questionnaire (BQ) and the Pittsburgh Sleep Quality Index (PSQI). Subsequently, the patients were examined using limited polygraphy, and the sensitivity and specificity of the questionnaires were evaluated. The STOP-Bang, Berlin and STOP questionnaires had the highest sensitivity for OSA detection (81.6%, 78.7%, and 74.2%, respectively), while the sensitivities of PSQI and ESS were low (50.8% and 34.5%). The ESS, STOP-Bang, STOP and Berlin questionnaires had the highest specificity (82.6%, 75%, 61.9%, and 61.9%). In our sample, we found the STOP-Bang and Berlin questionnaires to be the most suitable for OSA screening with the highest sensitivities (81.6%, 78.7%) and satisfactory specificities (75%, 61.9%). The STOP questionnaire was also relatively reliable, especially given its time-saving nature; though short, it preserved satisfactory sensitivity (74.2%) and specificity (61.9%). The ESS and PSQI were unsuitable for OSA screening.

## 1. Introduction

Obstructive sleep apnea (OSA) is the single most important preventable medical cause of excessive daytime sleepiness and driving accidents. OSA may also adversely affect work performance through a decrease in productivity and an increase in the injury rate. The odds of having a work-related accident were found to be nearly double in workers with OSA in comparison to controls [1]. An appropriate screening questionnaire for OSA could help identify high-risk workers and reduce the risk of accidents at work through therapy.

The severity of OSA is determined by the apnea hypopnea index (AHI) value (number of apneas/hypopneas per hour) and is divided into three grades of severity. An AHI range of 5–14.9 (with the presence of subjective difficulties) is indicative of mild OSA in the adult population, while patients with an AHI of 15–29.9 are considered to have moderate OSA, and those with an AHI of 30 and above are considered to have severe OSA.

The prevalence of obstructive sleep apnea (OSA) is estimated at one billion people worldwide, including over 400 million who have moderate-to-severe symptoms [2]. A number of screening methods for OSA exist: questionnaires, clinical screening models, and blood biomarkers to help identify patients with OSA [3,4,5,6,7,8,9,10]; however, until now, the gold standard for the diagnosis of OSA remains overnight monitoring performed by limited polygraphy (PG) or polysomnography (PSG).

This study aimed at comparing five established sleep questionnaires regarding their predictive probabilities for OSA: the Epworth sleepiness scale (ESS), STOP-Bang questionnaire, STOP questionnaire, Berlin questionnaire (BQ) and Pittsburgh Sleep Quality Index (PSQI).

## 2. Materials and Methods

### 2.1. Materials

In a prospective study carried out between September 2018 and March 2020, we examined a cohort of 237 consecutive patients in an outpatient clinic for snoring and sleep-disordered breathing at the ENT department. Patients were most often referred by a general practitioner, cardiologist, or an ENT physician. Some of them requested an observation following their partner’s complaints and/or their partners observing sleep apnea.

**Figure 1:**
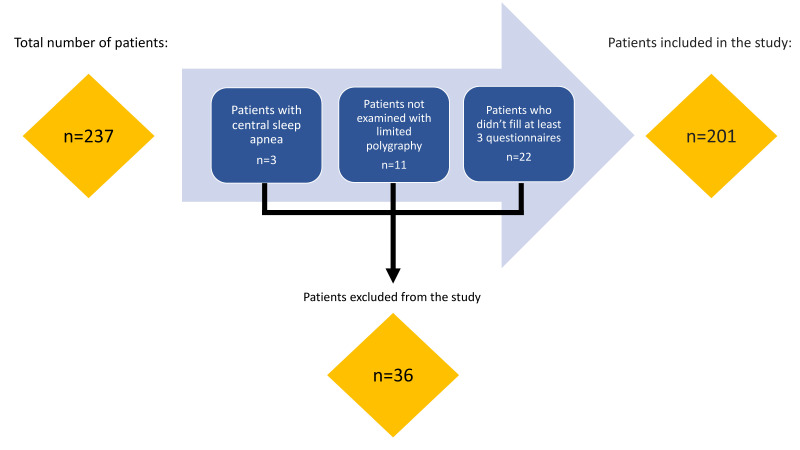
Flow chart—the inclusion/exclusion process.

Thirty-six patients were excluded from the study: three patients due to the presence of central sleep apnea, 11 patients that did not undergo a limited polygraphy examination, and 22 patients that did not complete at least 3 of the 5 questionnaires. A total of 201 patients were included in the study. We present the inclusion/exclusion process in Figure 1.

### 2.2. Descriptive Statistics and OSA of the Sample

A total of 143 men and 58 women were enrolled in the study. The mean age in years was 51.56 and the median was 52. The mean age was higher for women: 55, in contrast to 50 for men. The youngest patient was 19 years old, and the oldest was 75 years old. The mean and median BMI of patients were 30.9 and 30.5 kg/m^2^. The mean neck circumference in the patients was 41.8 cm, and the median 42 cm (for details, see Table 1).

In our sample, OSA was not present in 11.9% of the patients (AHI below 5). We found mild OSA (AHI 5–14.9) in 13.9% of the patients, moderate OSA (AHI 15–29.9) in 32.3% and severe OSA (AHI 30 and over) in 41.8% (for details, see Table 2).

### 2.3. Methods

Patients completed five written sleep questionnaires individually and were subsequently examined by limited polygraphy at the Department of Neurology. Manual polygraphy validation was performed.

BMI (body mass index)—defined as body weight divided by the square of height.

AHI (apnea-hypopnea index)—defined as the total number of apnea and hypopnea episodes in the course of 1 h.

Mild OSA—defined as 5 ≤ AHI < 14.9.

Moderate OSA—defined as 15 ≤ AHI < 29.9.

Severe OSA—defined as AHI ≥ 30.

### 2.4. Sleep Questionnaires Used in The Study

#### 2.4.1. The Epworth Sleepiness Scale (ESS)

The ESS was developed and validated by Johns [11] as a simple tool to assess excessive daytime sleepiness. The ESS consists of eight items that list various daily situations in which the patient evaluates the probability of falling asleep or napping using a scale of 0–3. The total score is the sum of the individual responses and is, therefore, in the range 0–24. Excessive daytime sleepiness and a greater likelihood of OSA are observed in patients with an ESS value > 10 [11,12]. In other studies, the sensitivity and specificity of the ESS vary, between 39–66% and 33–71%, respectively [13,14,15,16].

#### 2.4.2. STOP-Bang Questionnaire

The STOP-Bang questionnaire was developed by Chung et al. as a screening questionnaire for OSA [17]. It contains eight questions related to snoring, fatigue during the day, sleep apnea, high blood pressure, BMI, age, neck circumference and gender. It is possible to receive 0–1 points for each question. The total score is the sum of the individual answers and ranges from 0 to 8.

A score of 0–2 points indicates a low risk of obstructive sleep apnea (OSA), whereas 3–4 points indicate a medium risk, and 5–8 points indicate a high risk. A high risk can alternatively be indicated by a score of 2 for the first four questions plus BMI >35 kg/m^2^, or a score of 2 for the first four questions plus neck circumference (43 cm for men, 41 cm for women), or a score of 2 for the first four questions plus male gender.

Shrestha et al. found the sensitivity and specificity of the STOP-Bang questionnaire to be 92% and 33%, respectively. In a systematic review and meta-analysis by Bianca Pivetta et al., the sensitivity and specificity were found to be 91% and 28%, respectively. In the study by Costa et al., the sensitivity was lower, 68.4%, and the specificity was 85% [16,18,19].

#### 2.4.3. STOP Questionnaire

The STOP questionnaire is a simpler version of the STOP-Bang questionnaire. It was developed in 2008 in an attempt to establish an easy-to-use questionnaire for OSA screening in surgical patients [17]. It contains four questions about snoring, fatigue during the day, sleep apnea and high blood pressure. It is possible to receive 0–1 points for each question. The total score is the sum of the individual answers and is, therefore, in the range of 0–4. A high risk of OSA is indicated by a score ≥2. In the studies of Chung et al. and Patel et al., the sensitivity of the STOP questionnaire varied from 66 to 89% [17,20].

#### 2.4.4. Berlin Questionnaire (BQ)

The Berlin questionnaire was developed in 1996 at the Conference on Sleep in Primary Care in Berlin, Germany. It is a validated instrument that is used to identify individuals who are at risk for OSA in primary and some non-primary care settings. It contains 10 questions, which are divided into three categories. In the first category, there are five questions about snoring and breathing during sleep. In the second category, there are three questions about increased daily fatigue and drowsiness. In the last, third category, there are questions about hypertension and BMI. Each category is evaluated separately; the total score is calculated as the sum of points for each category and ranges from 0 to 3. A score of ≥2 indicates a risk for OSA [21,22]. Two previous studies found varying degrees of the sensitivity and specificity for the BQ: 73–83% and 22–44%, respectively [14,23].

#### 2.4.5. Pittsburgh Sleep Quality Index (PSQI)

The PSQI was not originally designed to screen for OSA. Rather, it is focused on sleep quality (sleep latency, sleep duration, sleep efficiency, sleep interruptions, use of sleep-inducing drugs, and daily dysfunction related to poor sleep) [24]. It contains 10 questions, which are divided into seven categories. Each category is evaluated separately using 0 to 3 points, and the total score is calculated as the sum of points for each category and ranges from 0 to 21. Poor sleep quality, which is also expected in patients with OSA, is noted for scores >5. The sensitivity of PSQI was shown to be low in two different studies (38–51%), and the specificity was shown to be 67–76% [16,25].

**Inclusion criteria:** (1) age over 18 years, (2) OSA assessment (diagnosis, follow-up) using PG, (3) completed three or more sleep questionnaires.

**Exclusion criteria:** (1) diagnosed with central sleep apnea, (2) OSA assessment performed using methods other than PG, or incomplete data from PG, (3) completed less than 3 sleep questionnaires, or questionnaires that were not answered completely.

### 2.5. Statistical Methods

Descriptive statistics (numbers, arithmetic mean, median, standard deviation, min. and max. value) were used to describe the data. Correlations between the results were evaluated using Spearman’s correlation coefficient. Furthermore, the sensitivity and specificity of individual screening questionnaires were evaluated. Statistical tests were evaluated at a significance level of 5%. The statistical program Stata version 13 was used for processing.

## 3. Results

For the Epworth Sleepiness Scale, 197 questionnaires were included and four excluded (for details, see Table 3). The sensitivity of ESS was 34.5%, and specificity 82.6%.

In the case of the STOP-Bang questionnaire scale, 183 questionnaires were included and 18 not included, with the best sensitivity of 81.6% and specificity of 75% (for details, see Table 4).

The STOP questionnaire scale had 184 included questionnaires and 17 not included questionnaires, with sensitivity of 74.2% and specificity of 61.9% (for details, see Table 5).

For the Berlin Questionnaire Scale, there were 185 questionnaires included and 16 not included, with the second-highest sensitivity of 78.7% and specificity of 61.9% (for details see Table 6).

The Pittsburgh Sleep Quality Index had 147 included and 54 not-included questionnaires, and had the worst results, sensitivity of 50.8%, and specificity of 47.4% (for details, see Table 7).

The highest sensitivity was found in the STOP-Bang questionnaire, the Berlin questionnaire, and the STOP questionnaire (81.6%, 78.7%, and 74.2%, respectively). The ESS and the PSQI had the lowest sensitivity (34.5% and 50.8%, respectively).

The ESS had the highest specificity (82.6%), followed by the STOP-Bang, STOP and Berlin questionnaires (75%, 61.9%, and 61.9%, respectively). The PSQI has the lowest specificity (47.4%) (for details, see Table 8).

## 4. Discussion

The aim of this study was to compare the predictive capabilities of five established sleep questionnaires for OSA. The questionnaires tested in this study were the ESS, BQ, STOP and STOP-Bang, as well as the PSQI. All questionnaires were filled in by patients presenting sleep disorders. The scores were evaluated against limited polygraphy based on AHI.

One of the most commonly used questionnaires in sleep medicine, the Epworth Sleepiness Scale, deals with only one of the presumed risk factors for OSA: excessive daytime sleepiness [11,26]. The advantage of ESS is clarity; it is a simple evaluation method. According to Johns et al., ESS scores significantly distinguished patients with primary snoring from those with OSA, and ESS scores increased with the severity of OSA [27]. However, the association between AHI and ESS scores was not confirmed by Laub et al. According to Laub et al., ESS is not a good questionnaire for the evaluation of the presence or severity of obstructive sleep apnea [28]. Similarly, in a study by Mediano et al., excessive daytime sleepiness measured by ESS was not invariably present in patients with OSA. Patients with OSA and excessive daytime sleepiness were characterized by worse nocturnal oxygenation than those without excessive daytime sleepiness. Both groups exhibited a similar AHI [29].

In other studies the sensitivity and specificity of ESS varied between 39–66% and 33–71% [13,14,15,16,30]. The results of our study demonstrated that ESS had a lower sensitivity for OSA (34.5%) and higher specificity (82.6%) in comparison to the findings by other authors. The low sensitivity was not surprising given that the ESS is a standard questionnaire designed to measure subjective excessive daytime sleepiness, which can occur secondary to multiple causes other than OSA.

The STOP-Bang questionnaire is widely used worldwide. [30] It is quick and simple. According to a meta-analysis by Chiu et al. from 2017, it had a high sensitivity (88%), but the specificity was low (42%) [30]. In an earlier study, it was found that the STOP-Bang questionnaire had high sensitivity for detecting moderate and severe OSA (93% and 100%, respectively), but the specificity of the STOP-Bang questionnaire was still low: 47% and 37% for moderate and severe OSA, respectively, resulting in fairly high false-positive rates [17]. Silva et al. reported that the STOP-Bang questionnaire had the highest sensitivity for moderate-to-severe (87.0%) and severe (70.4%) OSA in comparison to the ESS and the STOP [13]. In other studies, the sensitivity and specificity of the STOP-Bang questionnaire varied between 91–92% and 28–33% [16,18]. In our study, the sensitivity of the STOP-Bang questionnaire for OSA was found to be 81.6%, and its specificity 75%, which was higher compared to the study by Kee et al. (60% and 69%, respectively) [31].

The STOP questionnaire contains the first four questions from the STOP-Bang questionnaire. According to a meta-analysis from 2016, it had a sensitivity of 87% and a specificity of 42% [30]. In other studies, the sensitivity of the STOP questionnaire varied between 66 and 89%. In a systematic review article, Abrishami et al. recommended the use of the STOP-Bang and STOP questionnaires for their high-quality methodology and accurate results, although the sensitivity and specificity were not significantly higher compared to other questionnaires [32]. In our sample, the sensitivity of the STOP questionnaire for OSA was found to be 74.2%, and the specificity 61.9%. According to the results of our study, the STOP-Bang and STOP questionnaires were relatively suitable screening tools in comparison with other questionnaires.

The Berlin questionnaire is more time-consuming compared to the ESS, STOP-Bang and STOP questionnaires. Ahmadi et al. [33] tested the BQ with patients in a sleep clinic, retrospectively. Out of the 130 individuals tested, only 26.2% had a respiratory disturbance index (RDI) >10, whereas the BQ identified 58.5% as being at high-risk of having sleep apnea, with a 62% sensitivity and 43% specificity. The discrepancy between these results and our study could be attributed to the use of RDI rather than AHI at a higher cut-off (i.e., >10). In other studies, the sensitivity and specificity of BQ varied between 73–83% and 22–59%, respectively [14,23,30,31]. In our study, the sensitivity of the BQ for OSA was found to be 78.7%, and its specificity was established as 61.9%. Due to its satisfactory sensitivity and specificity, the BQ appears to be a suitable tool for OSA screening.

The PSQI is one of the most frequently used sleep questionnaires worldwide. Completing and evaluating the questionnaire is complex and time-consuming. The PSQI addresses psychological symptoms and correlates OSA with the occurrence of depression, anxiety or stress [34,35]. The PSQI is unsuitable for OSA screening. According to a study by Scarlata et al., the sensitivity of the PSQI was only 37.8%, and its specificity 76.1% [25]. In a different study by Amado-Garzón, the sensitivity for OSA and central apnea was 80–85% [36]. Based on our results, the PSQI had lower sensitivity in comparison to the STOP-Bang, STOP and BQ (50.8%,). The specificity was the lowest among all our questionnaires (47.4%).

A certain limitation of the study can be its monocentricity and the fact that not all patients filled in all five questionnaires completely. Patients that completed less than (or did not completely answer) three sleep questionnaires were excluded (see exclusion criteria). Another limit of the study could be the missing gender differences evaluation for the relatively small number of respondents (143 men and 58 women).

## 5. Conclusions

The STOP-Bang and Berlin questionnaires, which had the highest sensitivity (81.6%, 78.7%) and satisfactory specificity (75%, 61.9%), were found to be the most suitable for OSA screening in our sample. The STOP questionnaire was also relatively reliable, especially given its time-saving nature, which did not impair its satisfactory sensitivity (74.2%) and specificity (61.9%). The Epworth Sleepiness Scale and the Pittsburgh Sleep Quality Index had the lowest sensitivity (34.5%, 50.8%) and are unsuitable for OSA screening.

## Figures and Tables

**Table 1:** Basic indicators found in the whole group of patients (*n* = 201).

Indicators	Mean	Median	SD	Min	Max
Age (years)	51.6	52	12.32	19	75
BMI (kg/m^2^)	30.9	30.5	5.53	17.6	53
Neck circumference (cm)	41.8	42	4.33	20	57

SD—standard deviation.

**Table 2:** OSA in the sample group examined by limited polygraphy.

AHI	Total		Men		Women	
	Number	%	Number	%	Number	%
Total	201	100	143	100	58	100
<5	24	11.9	10	7	14	24.1
>5	177	88.1	133	93.0	44	75.9
Mild OSA	28	13.9	19	13.3	9	15.5
Moderate OSA	65	32.3	48	33.6	17	29.3
Severe OSA	84	41.8	66	46.2	18	31.0

**Table 3:** Results of Epworth Sleepiness Scale.

Epworth Sleepiness Scale (*n* = 197)					
Score	0–10	11–12	13–15	16–24	
	133	25	23	16	
	Mean	Median	SD	Min.	Max.
	8.3	7	4.69	1	22
PG	0–4.9	5–14.9	15–29.9	30–	
	23	28	63	83	

**Table 4:** Results of STOP-Bang questionnaire.

STOP-Bang Questionnaire (*n* = 183)					
Score	Low Risk	Intermediate	High Risk		
	45	68	70		
	Mean	Median	SD	Min.	Max.
	4.3	4	1.71	1	8
PG	0–4.9	5–14.9	15–29.9	30–	
	20	25	60	78	

**Table 5:** Results of STOP questionnaire.

STOP Questionnaire (*n* = 184)					
Score	Low Risk	High Risk			
	55	129			
	Mean	Median	SD	Min.	Max.
	2.2	2	1.16	0	4
PG	0–4.9	5–14.9	15–29.9	30–	
	21	25	60	78	

**Table 6:** Results of Berlin questionnaire.

Berlin Questionnaire (*n* = 185)					
Score	Low Risk	High Risk			
	48	137			
	Mean	Median	SD	Min.	Max.
	2.0	2	0.79	0	3
PG	0–4.9	5–14.9	15–29.9	30–	
	21	25	61	78	

**Table 7:** Results of Pittsburgh Sleep Quality Index.

Pittsburgh Sleep Quality Index (*n* = 147)					
Score	0–5	6–21			
	72	75			
	Mean	Median	SD	Min.	Max.
	6.4	6	3.5	1	19
PG	0–4.9	5–14.9	15–29.9	30–	
	19	21	47	60	

**Table 8:** Sensitivity and specificity of questionnaires.

Sensitivity and Specificity	ESS		BQ		PSQI		STOP Bang	STOP		
Test	Number	%	Number	%	Number	%	Number	%	Number	%
False neg.	114	65.5	35	21.3	63	49.2	30	18.4	42	25.8
True pos.	60	34.5	129	78.7	65	50.8	133	81.6	121	74.2
Total	174	100	164	100	128	100	163	100	163	100
**Sensitivity**	**34.5%**		**78.7%**		**50.8%**		**81.6%**		**74.2%**	
Test	Number	%	Number	%	Number	%	Number	%	Number	%
False neg.	19	82.6	13	61.9	9	47.4	15	75	13	61.9
True pos.	4	17.4	8	38.1	10	52.6	5	25	8	38.1
Total	23	100	21	100	19	100	20	100	21	100
**Specificity**	**82.6%**		**61.9%**		**47.4%**		**75%**		**61.9%**	

## Data Availability

Not applicable.
